# Ten simple rules for building an enthusiastic iGEM team

**DOI:** 10.1371/journal.pcbi.1009916

**Published:** 2022-03-04

**Authors:** Luis Garcia Morales, Niek H. A. Savelkoul, Zoë Robaey, Nico J. Claassens, Raymond H. J. Staals, Robert W. Smith

**Affiliations:** 1 Laboratory of Systems and Synthetic Biology, Wageningen University & Research, Wageningen, The Netherlands; 2 Scope Biosciences BV, Wageningen, The Netherlands; 3 Department of Social Sciences, Wageningen University & Research, Wageningen, The Netherlands; 4 Laboratory of Microbiology, Wageningen University & Research, Wageningen, The Netherlands; Carnegie Mellon University, UNITED STATES

## Introduction

**Fig 1:**
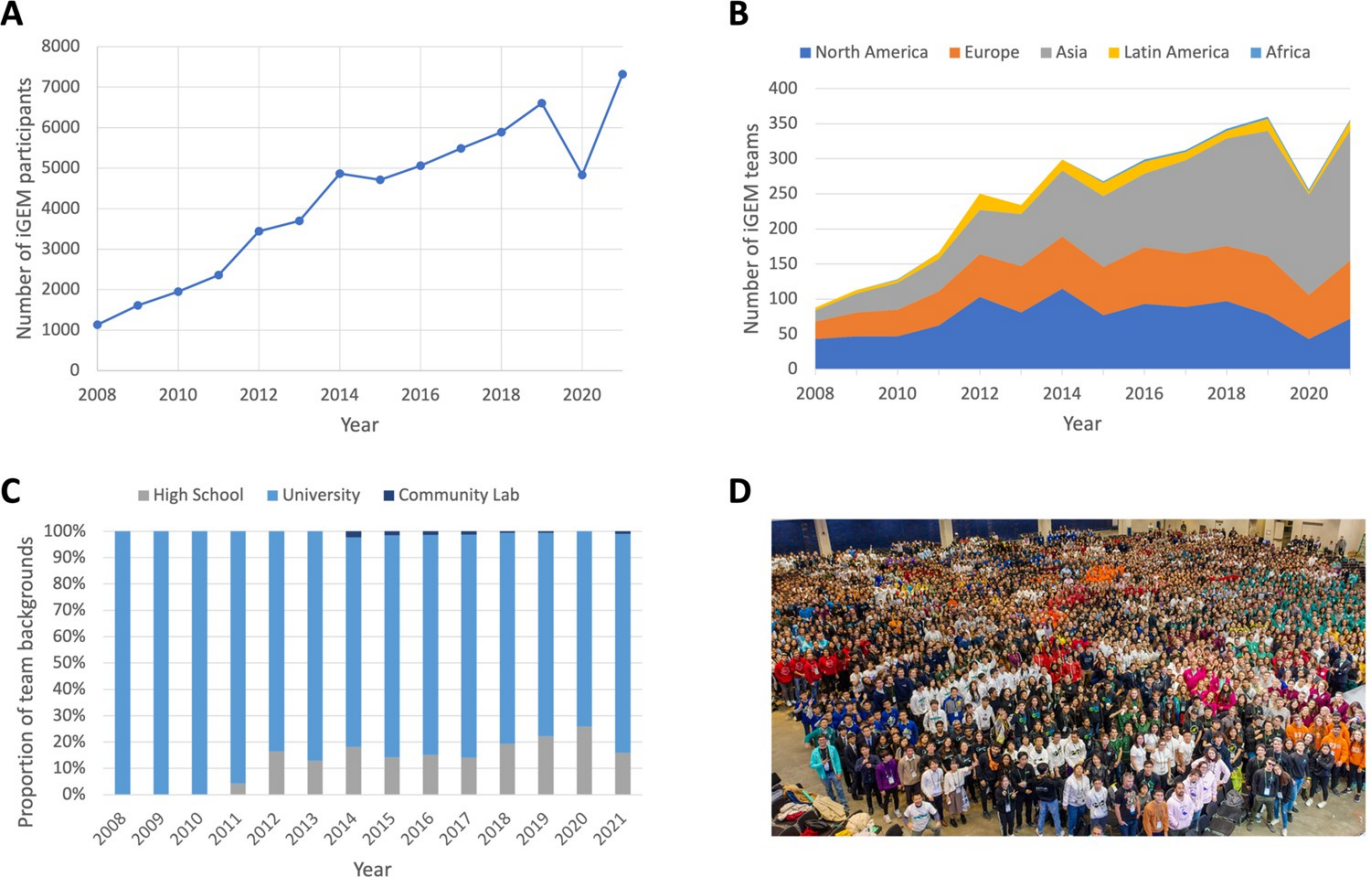
iGEM participation has increased around the world in the last decade. (A) Total number of recorded participants in iGEM competitions. (B) A breakdown of team locations for each competition. We see here a large increase in teams from the Asian region (as defined by iGEM) since 2014. In both instances, we see that the competition has recovered in 2021 after a COVID-disrupted 2020. (C) The background of teams shows an increase in high school entrants as a proportion of total entrants in the last decade. All data are publicly available from the iGEM Foundation. (D) Photo of the participants from the 2019 iGEM competition (photo from the iGEM Foundation). iGEM, international Genetically Engineered Machine.

Synthetic biology, as a research field, brings together molecular life scientists, computational biologists, and social scientists to (re)engineer biological systems toward societally desired goals. Given the field’s broad multidisciplinarity and relatively young age, innovative educational methods are required to provide students with the needed background knowledge to push the field forward in the future. The international Genetically Engineered Machine (iGEM) competition is such an example where education and high-level research merge, providing the synthetic biology field with trained students, new ideas, and novel results. In the 2021 edition alone, 343 teams from across the world completed the competition to tackle societal problems with synthetic biology (https://2021.igem.org; Fig 1). As Wageningen University & Research (WUR) celebrates 10 years of participation in iGEM, we share our thoughts and experiences on supervising iGEM teams, which is especially relevant to organizers of current and new teams, and also others interested in the iGEM competition.

iGEM originally started in 2003 as a course at the Massachusetts Institute of Technology (MIT) before turning into a competition in 2004 with 5 teams. Since then, the competition has continued to attract more participants—including high school teams (Fig 1C)—and incorporated more aspects of scientific research within the competition’s requirements. The key pillar of iGEM since its inception is that synthetic biology is used to solve societal issues. Further to this aim, teams are encouraged to also develop experimental tools that can be ported between different organisms and provide reproducible results across different laboratories so that projects can build upon one another [1]. Alongside experimental developments, the competition rewards teams that:

- use predictive modeling to design experiments;
- collaborate with other teams in the competition;
- consider the societal impact of their research and interact with stakeholders; and
- develop entrepreneurial plans to translate research into marketable propositions.

At the end of the competition, teams attend the Giant Jamboree where they can network with one another, present their work, and are appraised by a board of judges (Fig 1D). Based on a set list of criteria, the judges decide to what extent the team’s iGEM project can be categorized as a Bronze, Silver, or Gold medal–winning project. In addition, the judges also determine whether a project can be awarded competitive prizes—including the overall winner of the competition. In recent years, once the competition is over, participants can get involved in the “After iGEM” (https://after.igem.org) organization to maintain professional networks into the future, innovate later competition editions, and aid teams in their development.

As the number of requirements for iGEM teams has increased, so has the disciplinary diversity that is needed within each team. Successful teams comprise members capable of performing the abovementioned tasks. The competition is nowadays open to teams from a diverse range of educational backgrounds (Fig 1C). Here, we will focus our attention to the major category of participants: university teams and students. For many students across the world, there will not be many education programs available that can bring together such a wide range of topics. Alongside furthering their academic development, students participating in the competition develop a range of soft skills, including team organization, project management, scientific communication, fund raising, and advertising. As such, the competition-based education of iGEM goes beyond a normal academic curriculum, providing an intense training ground for students that wish to improve skills and broaden their horizons [2].

Over the last decade, at WUR, we have led teams that have researched methods to, for example, protect banana trees from infection (http://2014.igem.org/Team:Wageningen_UR), solve colony collapse disorder of bee hives (http://2016.igem.org/Team:Wageningen_UR), develop on-the-spot blood diagnostic tools (https://2017.igem.org/Team:Wageningen_UR), eradicate widespread plant pathogens (https://2019.igem.org/Team:Wageningen_UR), and tackle gas emissions from cattle (https://2021.igem.org/Team:Wageningen_UR). By researching these topics, our teams have finished second in the competition 3 times and won several other prizes along the way. We, the authors, have experience of participating in the competition as students, organizing and supervising multiple teams, judging entrants at the iGEM competition, and aiding iGEM with local event organization. Based on our experiences, and successes, we provide 10 rules for a successful iGEM team. We have split our 10 rules across 4 topics: developing a team (Rules 1 to 3), guiding the project (Rules 4 to 6), providing infrastructure (Rules 7 and 8), and celebrating your achievements (Rules 9 and 10). While we focus here on the iGEM competition, it is likely that many of these rules can be easily translated to other competition-based educational events, for example, DREAM for computational biology (https://dreamchallenges.org/) and BIOMOD for biomolecular nanotechnology (https://biomod.net). With this list, we hope to aid other iGEM organizers and teams, particularly those that have recently begun their iGEM adventure or that are considering entering teams in the near future, as the iGEM community expands to new areas.

### Rule 1: Assemble a complementary team for your multidisciplinary project

There are numerous ways in which an iGEM team can form. In some instances, teams will be formed from interested students that take the lead to find suitable scientific supervisors to guide their project. This was also the case with the first team from our institution (http://2011.igem.org/Team:Wageningen_UR). Currently at our institution, and likely at many others, iGEM teams are formed in a top-down manner where supervisors advertise the competition to a wide range of students. What is important, though, is that participating members are prepared to work within a highly multidisciplinary environment, as highlighted in the introduction. To build a strong team, it is beneficial that supervisors have experience in multidisciplinary research and that the participating students are aware and willing to further themselves in new disciplines. Individual supervisors and team advisors need not be experts of all fields—life sciences, mathematic/statistical modeling, computer science, or social science—but, rather, the aim should be to create a coaching group that can provide support in each domain. Likewise, it is ideally sought that students also come from a similarly diverse set of educational backgrounds. Of course, for a new team, such a broad pool of participants is unlikely to be found immediately. We would recommend focusing your search for participants in the life sciences. Experimental synthetic biology forms the backbone of iGEM projects and will allow students to expand their educational horizon to computational biology and scientific communication [2]. At WUR, we are fortunate that study programs provide students with the encouragement and opportunity to develop skills across a range of disciplines. For example, many of our students follow an MSc in Biotechnology or Molecular Life Sciences where experimental biology, mathematical modeling, and research ethics are all key educational tenets. To supply this education, researchers and teachers likewise span these domains at WUR, providing a large pool of potential iGEM participants.

Having taken part in multiple editions of the competition, we can build a group of supervisors for the student team based on PhD students and postdocs from the organizing departments, particularly those with previous experience or a strong interest in the iGEM competition. For new teams, or students looking for their own supervisors, we would recommend searching out departments at your institution with research expertise in synthetic biology and associated methods. The role of the supervisors is to provide advice to the team as the competition progresses, support their extracurricular activities, and supervise their research. Additional supervision can be sought out in humanities, social science, and even design departments: Chances are there is a scholar working on issues of synthetic biology closer than you think. On top of this, the iGEM foundation is also able to provide mentoring to newly created teams through an international network of ambassadors through “After iGEM”. It is useful for all teams to make use of available staff and ambassadors to understand how best to navigate and be successful in the competition.

**Fig 2:**
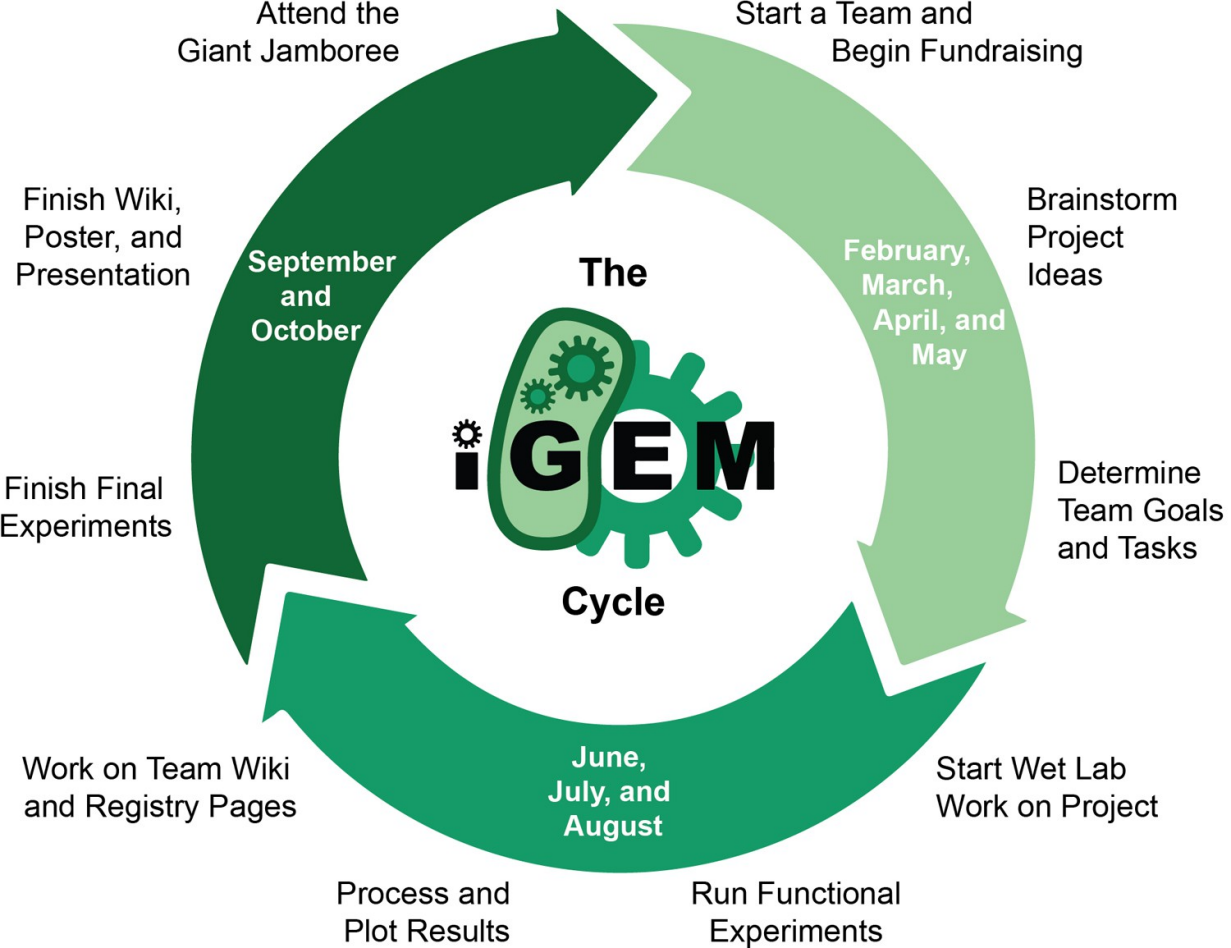
The iGEM cycle (2021). For a given calendar year, the iGEM competition begins around February with project development proposed to continue until May. From then on, the teams enter the “research phase” where they perform their experiments. Finally, in late summer to early autumn, teams start to prepare the deliverables for the competition. Picture produced by the iGEM Foundation. iGEM, international Genetically Engineered Machine.

Following our top-down organizational model, and with the supervision group in place, we then look to attract students to create a team for the competition. The construction process for an iGEM team tends to begin a few months prior to the competition officially beginning in March of a given year (Fig 2). This is to maximize the amount of time the team can spend on research when the competition begins. Our aim is always to have a final team of 10 to 12 students. While this team size is not a strict rule, we aim for this so that, first, students are able to produce enough research to fulfill educational requirements for their program during the competition (see Rule 2); and, second, we can effectively house the student team within our research facilities. Fortunately, we have never been short of interested students, nor needed to conduct a strict selection process—however, this could be considered if required, based on the student’s motivation or educational background. At an initial “kick-off” meeting, we will attract roughly 20 to 40 students, where they learn about the competition and see what previous teams have accomplished. The student–supervisor team then meets weekly to hear the project ideas students have before deciding on a final topic. Over the following weeks, we notice that the student team naturally decreases as students are drawn to other competing interests and commitments. What we are left with is a core group of dedicated students that are excited to develop their own synthetic biology project idea.

### Rule 2: Fit iGEM within your institute’s curriculum

Naturally, students have courses and requirements to fulfill to graduate from their program—this is the same for high school, undergraduate, and overgraduate iGEM teams (“overgraduate” teams contain MSc students). These include compulsory courses, completing a required number of credits, or conducting a thesis research project. If a student then takes part in the iGEM competition, this could result in 3 to 9 months—the typical time frame of iGEM projects—of extracurricular work on top of their education. It is often the time required for extracurricular work that leads to students pulling out of the iGEM team construction process discussed in Rule 1. As a result, we encourage team coordinators to consider ways of incorporating iGEM into a student’s curriculum such that students are rewarded for their efforts. This is likely to be particularly useful for institutions that regularly enter teams into the competition. We have found ways of doing this by offering students the opportunity to conduct their BSc or MSc thesis projects as part of iGEM. As such, the final iGEM project will consist of multiple thesis research projects knitted together. On top of this, we are also able to offer credit points to students via courses offered at the university that encourage multidisciplinary teamwork and project development. As such, from our example, taking part in an iGEM team does not have a negative effect on a student’s education timeline.

However, there are some aspects of the iGEM competition for which we are unable to offer full credit points, and, as such, the student team will still need to complete extracurricular duties [2]. These include conducting outreach activities (for example, presenting at conferences or interacting with stakeholders; see Rule 6), raising funds to perform research (for example, via sponsorship; see Rule 7), or building their iGEM wiki-style webpage (related to Rule 9). While it is critical for the success of an iGEM project, this extra workload should be kept in mind by the supervision team—as students are also understandably concerned about obtaining good grades and sufficient credits. At times, this can lead to a difficult balancing act of priorities and workloads for the team members that supervisors should help guide them through. We would encourage that supervisors guide the team to plan adequately for such activities and duties on top of their educational requirements and find ways to efficiently perform these tasks (see Rule 4).

### Rule 3: Aid the team with group organization to foster a positive working environment

Assuming that you have found a team of willing, enthusiastic, and dedicated students that wish to compete in iGEM, it is important to maintain an inclusive, creative, and positive team atmosphere. Between us, the authors, we have been involved in supervising multiple iGEM teams since 2014, and every year, each team is new, composed of different characters, and will require novel methods of management and organization. Of course, over the years, we have learnt from others and developed loose organizational rules, and we will summarize these here from a team and supervisor perspective.

From the team’s perspective, creating an appropriate organizational or management structure is important for success. In our experience, the following team roles are crucial:

- Team captain: key organizer of the team who maintains an overview of the project and team responsibilities. Also, they can act as a point of contact for the supervisors to discuss pressing organizational issues, team morale, and performance.
- Secretary: supports the team captain in collating agendas and minutes of meetings and managing communication within and without the team.
- Treasurer: keeps control of the team’s finances. This is vital for knowing how much money has been obtained from sponsors and can be spent on research, promotional material, team activities, and travel. Likewise, it introduces students to the importance of cost control when performing scientific research.
- Coordinator for outreach and human practices activities: finds, organizes, and conducts outreach activities on behalf of the team. This can include meeting with stakeholders, presenting the project at events, or making promotional material (for example, videos).

Of course, some of these tasks can be conducted by small teams, but it is important for the supervisors to know who the responsible people are for these activities. For example, it may make sense for the team captain to have help from another team member to maintain an overview of project planning, or for the coordinator of outreach activities to lead a small team. However, we would advise against creating too many niche individual roles, as this can lead to the dilution of responsibility and a lack of teamwork between team members.

It is these final points related to team cohesion that supervisors should keep aware of. Over the course of a 3- to 9-month time span, in a competitive research environment, undesired conflicts between team members may occur (as noted in recent team surveys [3]). Supervisors should try to identify these (in communication with the team captain) at an early stage and motivate the team as a whole or the individuals involved to find a resolution by, for example, creating codes of conduct. Tighter bonds within the team will form if they are positively encouraged to find their own solutions and to navigate bumps in the road independently.

### Rule 4: Direct your student team toward feasible project designs

In the rules above, we have focused on how to find participants for your iGEM team and how we suggest they are organized. It is also important to remember and recognize that many participants will not have previously been a part of a multidisciplinary research team or had to organize their own project. Therefore, it is important for the lead supervisors to provide a framework for the students to organize their meetings, develop their thoughts, and build their team.

During the initial meetings with interested students—as your team begins to form—it is important for the supervisors to lead conversations and set the agenda for discussions. The supervisors should help set schedules for meetings, provide initial timelines for the team so they know when deadlines need to be met, and introduce concepts such as GANTT charts to help the team design and find a good topic for their iGEM project. The first key milestone for the team is to settle on 1 or 2 feasible project ideas that could be successful in the competition.

**Fig 3:**
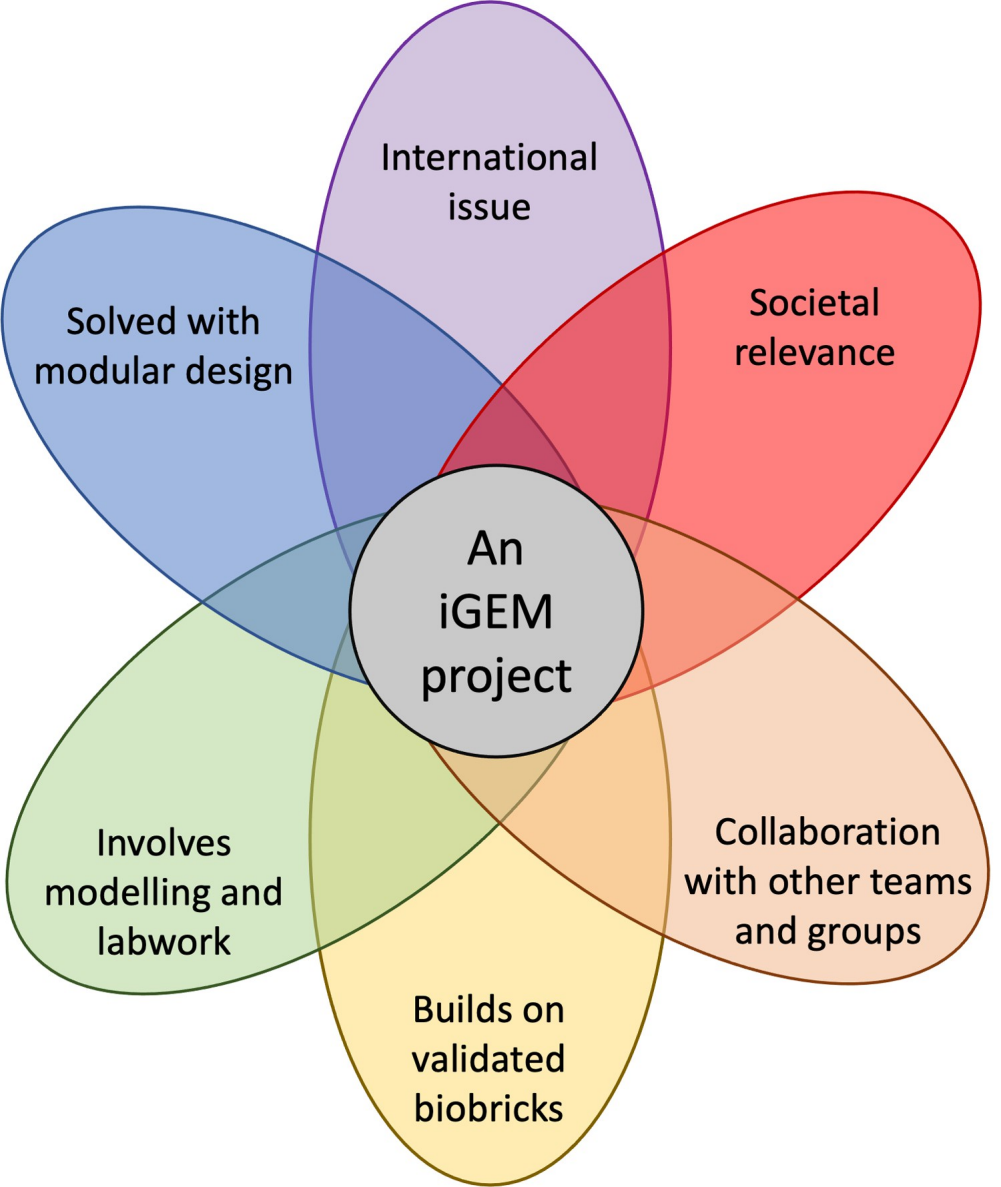
The 6 tenets of our ideal iGEM project. The project should aim to tackle an international problem that can involve local stakeholders. The project should be modular in design such that students can “own” individual subprojects, and should allow for a seamless interaction between experimental and computational biology. By building the project on already available iGEM materials, the project can then allow for easy collaboration with other teams and further develop the iGEM foundation’s parts repository. iGEM, international Genetically Engineered Machine.

At WUR, we construct iGEM projects around 6 key tenets (Fig 3). The project:

- provides a synthetic biology solution to a real-world issue;
- stimulates public interest in cutting-edge scientific research;
- develops on synthetic biology tools previously developed for iGEM (referred to as BioBricks; for published examples, refer to [1,4,5]);
- allows for potential collaboration opportunities with other iGEM teams through the development of tools or testing synthetic constructs;
- incorporates mathematical modeling to aid system and experimental design; and
- can be divided into submodules such that each member of the team can research an individual subproject.

If a project idea meets the above criteria, the student team can then move to the next stage and check if the project can feasibly lead to substantial results within 6 months of research. One possible methodology for this is to create subteams dedicated to “dry lab,” “wet lab,” and “human practice” research; however, we have found that intertwining the 3 areas at the project design stage successfully stimulates multidisciplinary research [3]. Note that the project topic does not need to match the exact research expertise of the involved supervisors. We do not believe this is a requirement for a good iGEM project, and students should be allowed to pick a topic that is of interest to them. This can lead to a rewarding experience for the supervisors as they are forced to leave their comfort zones and learn something new alongside the students. The team can also benefit from this strategy as they need to search for external support and expert insights from stakeholders—a critical aspect of the iGEM competition.

Through the course of the project refinement process, students will naturally develop and find roles for themselves within the group based on their predispositions. For example, captains will be more vocal, lead discussions, and find consensus among the different opinions of the team. As the team dynamic evolves, the supervision group should allow the team to take on more responsibility in leading and organizing meetings. Once the project begins, such independence will be required by the team (see Rule 5) as supervisors increasingly take on a coaching/advising role.

After this initial phase of iGEM, you should have a dedicated and organized team of students, along with 1 or 2 excellent, feasible iGEM project ideas. In instances where multiple projects have been found, we ultimately decide the final project idea democratically, with every student and supervisor having a vote in the final decision. For example, when conducting such a vote, project preferences could be based on iGEM criteria of previous years. Ultimately, the students will feel that they “own” their project.

### Rule 5: Coach your team as they gain more independence

**Fig 4:**
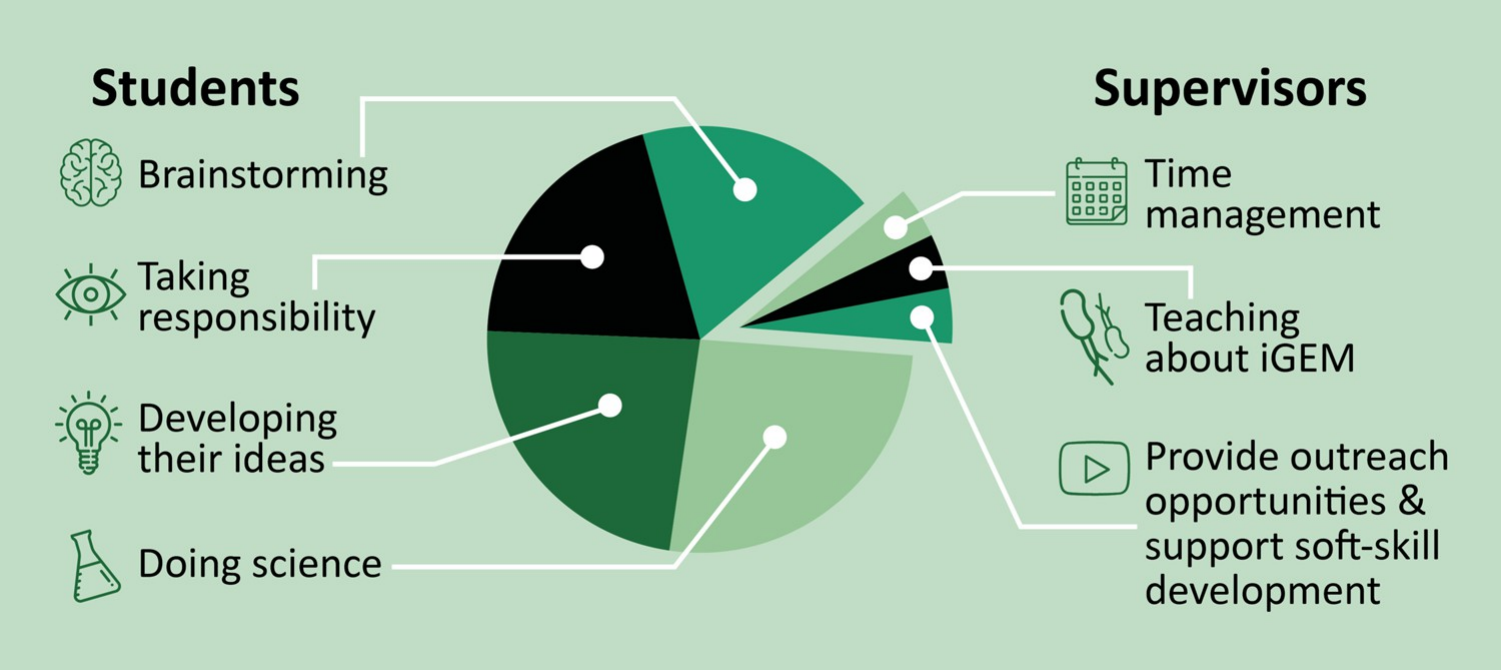
Task division between students and supervisors. The role of the supervision team is to coach the students to perform research. As such, much of the “heavy work” in relation to project design, conducting research, and interacting with stakeholders need to be performed by the students. Icons were made with Freepick, DinosoftLabs, Srip, and Smashicons from Flaticon. iGEM, international Genetically Engineered Machine.

With a team and project in place, it is now over to the students to execute the research, reach out to stakeholders, and start to build toward the Giant Jamboree. The students should now take over from the supervisors to organize group meetings, set agendas, and provide timelines for their project—this is a key task of a team captain. Supervisors should be on hand to provide support, advice, and help troubleshoot issues the team may come across during their research (see Fig 4). This is most likely to be required at 2 levels: scientific issues and competition requirements.

In the first instance, it is important to remember that the student team will be relatively inexperienced with scientific research. In many institutions, students will learn basic methodological practices in courses and may have already completed a research project as part of an earlier education program, but the students will still require general troubleshooting help regardless of the project topic. Common issues often include plasmid assembly, genome editing, or development/simulation of mathematical models. On top of these day-to-day technical problems, the team may also require help in project management. Given the restricted time provided by the competition environment, it is important to always keep in mind that a final, well-rounded research story needs to be delivered at the end of the project. As such, students may require help in maintaining a “helicopter overview” of how subprojects fit together, and whether they will be feasible within the limited time period they have available to them. Coaching the students toward effective, pragmatic solutions and project designs is a key responsibility of the supervisors.

In the second instance, and as stated above, iGEM is a competition. As such, there are requirements and milestones teams need to reach to claim awards and win prizes. The best examples of this are the requirements to receive a “Gold medal” and to organize the team webpage/wiki prior to the “Wiki freeze” deadline. If a team achieves “Gold medal” status, they are likely to be considered for further “Best of” category awards, as decided by a board of judges. To obtain this status, the team needs to complete the required activities for Bronze and Silver medals, and then provide evidence that they have completed at least 3 Gold medal requirements. To help the students navigate a path toward their desired target, the student–supervision group should check the year’s requirements and discuss which ones can be achieved, with supervisors suggesting possible paths to achieving these goals based on previous experiences. Likewise, when it comes to ensuring that the work is present on the wiki-style webpage ahead of the competition deadline, supervisors should be on hand to provide advice and read draft documents. From our experiences, it is important to note that we are not telling the students explicitly how to achieve goals, we advise the team toward pragmatic ideas, and we trust the team to achieve the goals they set for themselves. By entrusting the team to lead themselves, they will gain confidence in their independence and will, arguably, learn more about being a scientist as compared with more hands-on supervision.

### Rule 6: Support soft skill development and taking outreach opportunities

One of the great aspects of the iGEM competition is the ability to give students well-rounded training experiences. To be successful in the iGEM competition requires teams to develop their soft skills. These are often not taught during education programs, and, as such, coordinators and supervisors should allow students to have the time to perform these activities and get training where required. Typically, these are activities that are not necessarily part of the curriculum (Rule 2). Here, we will highlight 3 examples of outreach that we encounter in each edition of the competition: science communication, collaboration, and outreach.

The first example centers on scientific communication. Within the competition, teams are judged on so-named “integrated human practices”—the process by which stakeholder interactions can help influence, design aspects of, and appraise the impact of a scientific project. It is important to recognize that students have, likely, not been trained in communicating scientific research with other students, the public, or important stakeholders before. These activities require the development of communication skills as a two-way street, where students are not only able to effectively communicate complicated topics with different audiences, but also listen to these audiences’ concerns, experiences, and knowledge. As such, the team should be encouraged to train in these aspects either by utilizing help from experts within the host institution (if available) or by being offered presentation opportunities by the supervisors (for example, by presenting lectures or conducting hands-on practical classes). Supervisors should also be aware that pursuing such activities will lead to a decrease in lab or modeling research productivity for a few days. This should be allowed as it gives the opportunity for other kinds of investigations that are just as relevant to their project.

Collaboration is the second example we focus on. The iGEM competition, through its medal requirements (Rule 5), encourages teams to meet and collaborate with one another. Team collaboration is an important part of the competition and requires sharing, debugging, or testing research performed between teams. In recent editions, the competition has included a “Partnership” category where teams collaborate more often throughout the entire competition cycle. The benefits of the collaboration requirement are 2-fold. First, teams can share their experiences with like-minded others and could lead to the start of a scientific network that survives over the years. Second, having teams test the reproducibility of experimental parts and computational pipelines/models helps the iGEM Foundation create a library of tools to be used within scientific research—the academic outcomes of which are now seen in peer-reviewed synthetic biology literature [1,4–6].

The third example we discuss is outreach. Each iGEM team needs to generate funds for their project (see Rule 7), and the best way to do this is to advertise via social media, blogs, crowdfunding initiatives, presentations, videos, and through host institutions webpages and news outlets. Many students are savvy social media users—we have, for example, tried Instagram karaoke and competition-themed initiatives—to generate connections with other teams and companies. Likewise, if a press release can gain traction, then individual iGEM teams have been known to get national and international recognition. While media activities are not formal requirements for the iGEM competition, they again require time and training. However, video production will likely form a key part of the iGEM competition in coming years, and this should be considered from the start of the competition. Finding ways to combine activities or share content over multiple platforms is a useful way of efficiently promoting the team via media forms without placing too much work pressure on the student team at one point in time.

### Rule 7: Provide infrastructure and gift a financial seed to kick-start the project

As discussed above, teams need space to conduct research, meet with each other, and plan or conduct a range of activities. Therefore, it is important to provide facilities for these activities. For example, office space where the team can work together on extracurricular activities and conduct “dry” research, as well as meeting rooms for larger gatherings with the supervision team and, most importantly, lab space for experimental research. Such shared spaces are vital for team building and team cohesion, as the students balance their research with more social aspects of teamwork. At WUR, we are fortunate to have access to such facilities within our departments and around the university campus. We are aware that other iGEM teams will not be so fortunate. While the lack of such facilities does not stop teams from producing high-quality iGEM projects, supervisors and administrators can take a large burden away from the student team by providing such facilities. This allows the team to focus on conducting research, and all other fundraising, and outreach activities demanded by the competition.

Along similar lines, iGEM projects are often not cheap endeavors and will be the first time students fully grasp the costs of performing scientific research. Although this will differ with location and currency, we estimate the total costs and aim to raise in the region of €35,000 to €40,000 each year, in line with estimates made by others [7]. This budget covers the scientific research, competition registration fees, travel and accommodation costs during the final Giant Jamboree, and other related costs such as producing promotional material or team sweaters. Due to the costs needed, our organizing departments supply our team with a seed fund of roughly €5,000 to €10,000 at the start of the competition to get their project up and running, a practice followed at other universities such as in Canada [3,7]. This reduces the burden on the team to obtain start-up costs as they prepare advertisements and contact potential sponsors (for example, companies and other funds) to obtain further financial aid.

### Rule 8: Build and utilize an iGEM network

We hope we have illustrated with Rules 1, 4, 6, and 7 that it is useful for team coordinators and supervisors to have a range of contacts, both within their host institution and with the wider iGEM community. While we appreciate that such a network takes time to build, a supervisor’s connections can help find sponsorship funding, provide outreach opportunities to the team, or aid in media training and advertisement. Furthermore, for new supervisors, iGEM offers support for team development (see below). By providing a team with a list of contacts (potentially utilized by previous teams), the search for sponsors and outreach activities can begin at the start of the project, rather than losing time and opportunities by searching for novel contacts. In our experience, each project requires new contacts and new stakeholders (due to project topics changing year to year), but many sponsors and contacts are willing to offer a helping hand to multiple teams. Over the years, we have maintained and added to a list of sponsors, and we have developed a network of contacts within our host institution that can aid with financial management, social media training, and press releases.

Of course, a supervisor’s personal scientific network can also be used during an iGEM project. This could be to help obtain a certain bacterial strain for an experiment or find experts within a given field to supervise a student’s subproject. By adding a supervisor’s name to communications between team members and another scientist or contacting the scientist on the team’s behalf, the collaboration process can be accelerated. In a similar fashion, if the project topic is outside of the supervision team’s expertise (see Rule 4), then asking colleagues and collaborators within your host institution for advice can also be accelerated through direct contact rather than indirectly through the team. We have found the benefits of helping the team utilize our networks multiple times throughout the years and have collaborated on student supervision with multiple scientists within WUR.

The paragraphs above assume that the supervision team have already organized multiple iGEM teams. However, even for newly formed teams, support networks are in place for students and supervisors to provide training and scientific advice. The iGEM Foundation, who organize the competition, have successfully put together a network of iGEM ambassadors (https://after.igem.org/ambassador-program) around the world that are on hand to support teams and offer related training or advice. The ambassadors are often previous participants in the iGEM competition, and current participants can also stay involved in the organization, maintaining a professional network via the “After iGEM” initiative (https://after.igem.org). Utilizing such iGEM networks can be incredibly useful to help set up new teams and guide new supervisors through the iGEM competition. Furthermore, iGEM HQ is comprised of several committees of experts who are available to answer teams’ queries (https://igem.org/People/Committees). For new supervisors, it can also be especially beneficial to join your team at the final Giant Jamboree, meet other scientists and supervisors involved in the competition, and those that judge the final products of teams at the competition. This can help expand your personal network and provide good insights for future editions of the iGEM competition. Between the authors of this paper, we have attended multiple iGEM jamboree events, helped judge teams, have participated in iGEM committees, and are involved in iGEM ambassador networks. As such, we can endorse that maintaining links with the iGEM foundation can help direct your team toward a successful competition strategy.

### Rule 9: Prepare and perfect the students for the final jamboree

If you have successfully navigated the hurdles illustrated above, then your team will be racing against the clock to complete their research ahead of the late October “wiki freeze”. The “wiki freeze” is the deadline by which time all aspects of an iGEM project need to be posted on the team’s webpage. After this deadline, it is no longer possible to edit or post new information as it is these webpages that judges view to assess the teams. After the webpage has been frozen, the team needs to prepare for the Giant Jamboree. The jamboree is, arguably, one of the biggest international synthetic biology conferences, bringing together thousands of student participants, supervisors, scientists, and judges. Across a packed schedule covering several days, teams will have to present their work in front of an audience of judges and participants, be quizzed by judges at their poster stand, demonstrate prototypes of tools and software created during the project, and, of course, enjoy social events with other iGEM participants. The culmination of the event is a large closing ceremony where teams learn if they have received medals, been nominated for “Best of” awards, or been voted the best project in the competition.

Given that the competition does not end at the “Wiki freeze” and that students are not likely to have experienced a conference environment before, it is important to prepare the team appropriately. Historically, the 2 most important aspects of the Jamboree are the oral and poster presentations, both of which are assessed by a panel of judges. In recent online editions (due to the COVID pandemic), the focus has switched to video production and an oral defense of the research under examination by judges. Regardless of the requirements, our strategy has often been to divide the team into subgroups (for example, oral presentation and poster design), with an assigned set of supervisors for each task so that the workload is equitably distributed and deadlines are agreed among the team. The aim of the poster team is to create an informative scientific poster, aided by an appealing environment around the poster stand, to attract visitors and advertise the project presentation later in the Jamboree event. The goal of the presentation team is to construct a fluid, concise story from all of the research and human outreach conducted during the competition. Given the limited time for the presentation, it can often be difficult to discuss every aspect of performed research within the presentation, and that should not be the aim. From our experience, presenting fewer results around a high-quality story that brings together experimental synthetic biology, mathematical modeling, and integrated human practices, will be more impactful to the audience and judges. For a video presentation, we have followed a similar such strategy. On top of helping the students practice telling their research “story,” it is also useful for supervisors to help the team prepare for questions and defend their work. As with all scientific presentations, entering into scientific debates and defending research is equally important as presenting the work. This, again, is something that students are unlikely to have experienced before. Therefore, providing training time for this to the team can be beneficial for success in the competition. It is important to note that judges will typically visit the posters and evaluate students’ engagement in scientific discussions.

Outside of these competition requirements, it is important for the supervision team to recognize that the Jamboree is also the culmination of an intense research project for the students. As such, the students should be encouraged to relax, explore, and enjoy themselves. We invite our teams to find a way to stand out from the crowd—whether that be by wearing outrageously colored sweaters, have their own mascot running around the jamboree, or by giving out small, fun pieces of promotional material to other participants. This all adds up to the team attracting a lot of attention, meeting lots of new people, and enjoying a fun experience. To help oversee this, we send 2 to 3 supervisors with the team that can aid the team in networking and talking with other scientists. It is important to recognize that students can really benefit from connections they make at the iGEM jamboree, which could result in them obtaining unique internship experiences, future research positions, or roles within the iGEM Foundation. Your student team should be encouraged to take advantage of the opportunities presented at the Jamboree as much as possible.

### Rule 10: Celebrate generously

It is the end of another iGEM cycle, your team has researched a novel topic, planned and carried out the multidisciplinary tasks required, interacted with stakeholders, and obtained funding. They have gone to the Jamboree, represented your institution with pride, excited the audience with their work, and received recognition from the judges. The final step is to celebrate.

With your support and help, regardless of the results from the competition, it is almost a guarantee that the students have gone further scientifically than you—the supervisor—would have thought possible at the start of the project. Once the Jamboree has finished, allow the team their chance to enjoy themselves at the closing ceremony or take a holiday in the days after. Upon their return to your institution, a more formal celebration can be organized that recognizes the team’s achievements. Ultimately, and the main point we wish to emphasize here, is that iGEM is centered on the student’s achievements and development as the future generation of synthetic biologists.

Once the celebrations are over, it is time for the team to go on to the next stage of their education programs or graduate and move on to new pastures. For the supervision team, it is time to return to Rule 1 and build a new team for the next iGEM cycle having learnt from the experiences of the previous year. For the student team, they may wish to try and publish some of their research results or develop their project into an entrepreneurial venture [2,6]. However, even if the project is not continued, there is a good chance that the paths of supervisors and team members will cross again. From our experience, the students that form and complete an iGEM project go on to find PhD positions in the future and may even come back to the iGEM competition as part of supervision teams. To our minds, therefore, iGEM provides a fantastic snapshot of future career potential to synthetic biology students who are willing to go the extra mile and reap the benefits as a reward.

## Conclusions

The iGEM competition provides high school, undergraduate, and overgraduate (MSc) students, as well as community members who are interested in synthetic biology, with a full research experience. From spotting an unsolved problem, coming up with a scientific solution to the issue, then reaching out to the public and raising funds to perform research, the complete research cycle is tested within the competition. The students, though, are not in this alone and will require the support and organization provided by a team of scientifically experienced supervisors. In the 10 rules presented above, we have tried to show readers how we, at WUR, help teams through an iGEM cycle. Our overriding hope, by presenting these rules, is that current team leaders may learn something new from our experiences and ideas or that scientists (students or supervisors) new to the iGEM competition will be inspired to create a new team.

What we hope we have illustrated above is that the iGEM competition is a fascinating experience for both students and supervisors. The competition is always growing and evolving. As such, we would like to provide 2 perspectives for the future. First, in part due to the pressures brought by the COVID pandemic, the competition requirements have evolved to include developing information videos for projects, alongside classical presentations and posters. This is a further skill that students will need to develop and is not usually taught in other parts of education programs. However, it would be interesting to see if this technological development also percolates out of iGEM and becomes a common means of presentation for scientific conferences in the future and, as such, a skill we will all need to develop over the coming years. Second, as suggested previously, we would encourage the iGEM community to consider the costs involved in hosting a successful iGEM team [7]. As the competition expands, and more project criteria are expected of teams, more finances are required. Limiting the costs required to enter the competition or providing easier access to willing sponsors would make it much easier for new teams to enjoy the competition as we have done for a decade.

Finally, we would like to point out that throughout our 10 rules, we have sparingly referred to the fields that make up the iGEM competition: experimental synthetic biology, mathematical modeling, and social science. This is purposeful, as we believe our advice can be equally applied to other multidisciplinary competitions. The role of the supervisor is to help coach the team’s organization and research activities, link the competition with a student’s education program, encourage soft skill development, and find ways to advertise the project as broadly as possible. As such, although we have focused on iGEM, we hope that coordinators of student teams from other research competitions can take something away from these rules with the hopes of (1) leading a successful team; and (2) producing well-rounded high-quality students that will be our future academic, policy, and industrial leaders.
